# ERS International Congress 2023: highlights from the Basic and Translational Sciences Assembly

**DOI:** 10.1183/23120541.00875-2023

**Published:** 2024-04-29

**Authors:** Karosham Diren Reddy, Nikoleta Bizymi, Anja Schweikert, Sachin Ananth, Clarice X. Lim, Katharine M. Lodge, Audrey Joannes, Niki Ubags, Anne M. van der Does, Suzanne M. Cloonan, Arnaud Mailleux, Nahal Mansouri, Niki L. Reynaert, Irene H. Heijink, Sara Cuevas-Ocaña

**Affiliations:** 1 Epigenetics of Chronic Lung Disease Group, Forschungszentrum Borstel Leibniz Lungenzentrum, Borstel, Germany; 2 Division of Pediatric Pneumology and Allergology, University Medical Center Schleswig-Holstein, Lübeck, Germany; 3 Laboratory of Molecular and Cellular Pneumonology, School of Medicine, University of Crete, Heraklion, Greece; 4 Department of Medicine, Royal College of Surgeons in Ireland, Dublin, Ireland; 5 London North West University Healthcare NHS Trust, London, UK; 6 Institute of Medical Genetics, Center for Pathobiochemistry and Genetics, Medical University of Vienna, Vienna, Austria; 7 Ludwig Boltzmann Institute for Lung Health, Clinic Penzing, Vienna, Austria; 8 National Heart and Lung Institute, Imperial College London, London, UK; 9 Université de Rennes, CHU Rennes, Inserm, EHESP, Irset (Institut de recherche en santé, environnement et travail) – UMR_S 1085, Rennes, France; 10 Division of Pulmonary Medicine, Department of Medicine, Lausanne University Hospital (CHUV), University of Lausanne (UNIL), Lausanne, Switzerland; 11 PulmoScience Lab, Department of Pulmonology, Leiden University Medical Center, Leiden, The Netherlands; 12 School of Medicine, Trinity Biosciences Institute, Trinity College Dublin, Dublin, Ireland; 13 Université Paris Cité, Inserm, Physiopathologie et épidémiologie des maladies respiratoires, Paris, France; 14 Department of Respiratory Medicine and School of Nutrition and Translational Research in Metabolism, Maastricht University Medical Centre, Maastricht, The Netherlands; 15 University of Groningen, University Medical Center Groningen, Department of Pathology and Medical Biology, Groningen Research Institute for Asthma and COPD (GRIAC), Groningen, The Netherlands; 16 University of Groningen, University Medical Center Groningen, Department of Pulmonary Diseases, Groningen Research Institute for Asthma and COPD (GRIAC), Groningen, The Netherlands; 17 Biodiscovery Institute, Translational Medical Sciences, School of Medicine, University of Nottingham, Nottingham, UK; 18 These authors contributed equally

## Abstract

Early career members of Assembly 3 (Basic and Translational Sciences) of the European Respiratory Society (ERS) summarise the key messages discussed during six selected sessions that took place at the ERS International Congress 2023 in Milan, Italy. Aligned with the theme of the congress, the first session covered is “Micro- and macro-environments and respiratory health”, which is followed by a summary of the “Scientific year in review” session. Next, recent advances in experimental methodologies and new technologies are discussed from the “Tissue modelling and remodelling” session and a summary provided of the translational science session, “What did you always want to know about omics analyses for clinical practice?”, which was organised as part of the ERS Translational Science initiative's aims. The “Lost in translation: new insights into cell-to-cell crosstalk in lung disease” session highlighted how next-generation sequencing can be integrated with laboratory methods, and a final summary of studies is presented from the “From the transcriptome landscape to innovative preclinical models in lung diseases” session, which links the transcriptome landscape with innovative preclinical models. The wide range of topics covered in the selected sessions and the high quality of the research discussed demonstrate the strength of the basic and translational science being presented at the international respiratory conference organised by the ERS.

## Tweetable abstract

**In case you missed the #ERSCongress 2023, this article highlights key messages of the @EuroRespSoc @3Assembly (Basic and Translational Sciences) sessions that were discussed in Milan! @SaraOcana1 @EarlyCareerERS @ERSpublications** https://bit.ly/3MO0eII

## Introduction

The European Respiratory Society (ERS) International Congress 2023 took place in hybrid form, hosting more than 20 000 participants who attended either in person (17 309 registrations) in Milan, Italy, or online (3215 registrations). The congress focused on tackling key areas of respiratory medicine: pollution, climate change and sustainable developments. As in previous years [1–3], there were numerous types of sessions including oral presentations, symposia, hot topics, poster presentations and year in review [4]. In this article, early career members of ERS Assembly 3 (Basic and Translational Sciences) [5] summarise some of the most relevant sessions describing the latest state-of-the-art technologies and sessions giving insights into the future direction of basic and translational respiratory science [6]. Additional content can be accessed on the virtual platform (https://channel.ersnet.org/programme-live-161) and abstracts at https://erj.ersjournals.com/content/62/suppl_67.

## Oral presentation session: Micro- and macro-environments and respiratory health

**FIGURE 1:**
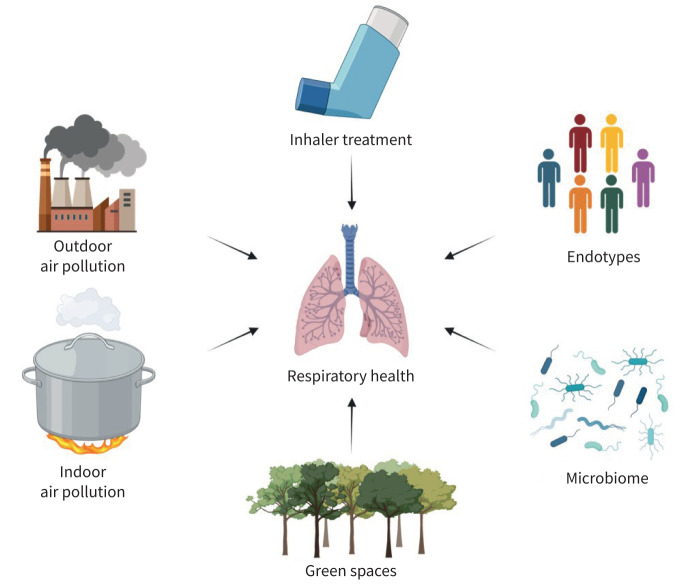
Schematic of the discussed environmental factors that affect lung function, including the microenvironment level of pathophysiological changes (endotypes) and microbiome, the macroenvironment level of indoor and outdoor air pollution and green spaces, and the use of inhaler treatment. Created with BioRender.

The respiratory system is closely linked with the environment. Various elements in the surroundings affect lung function, ranging from endotypes, the microbiome and the microenvironment up to the macroenvironment, including indoor and outdoor air pollution and green spaces. This also encompasses the use of inhaler treatments. These factors interact with one another within a complex network, influencing respiratory health as assessed by spirometry measurements such as forced vital capacity (FVC), forced expiratory volume in 1 s (FEV_1_), fractional exhaled nitric oxide (*F*_ENO_), symptom burden, allergic rhinitis, COPD and the incidence of asthma (figure 1).

Backman and co-workers [7, 8] showed that COPD endotypes can be identified through different lung function trajectories with unique biomarker profiles. Three trajectories (T1: mean age 65 years, ever-smokers 72%; T2: mean age 58 years, ever-smokers 100%; T3: mean age 71 years, ever-smokers 78%) were described that exhibit combinations of different values of FEV_1_, mean high-sensitivity C-reactive protein, matrix metallopeptidase (MMP)-9 and MMP-9/tissue inhibitor of metalloproteinase (TIMP)-1 ratio [8, 9]. Cornu Hewitt *et al.* [10] presented a study assessing the association between livestock-related emissions (*e.g.* bacteria and antimicrobial resistance genes) and the structure of the oropharyngeal-acquired resistome (defined as an inherited set of genes used to resist infections) in COPD individuals *versus* healthy individuals. This study showed that the airway of individuals with COPD exhibited higher resistome diversity, while *Escherichia coli* was associated with significant differences in the oropharyngeal resistome of all individuals in the study [11–14]. R. Bertelsen (Bergen, Norway) explained the link between the exposure to indoor bacteria and lung function and inflammation in children. More specifically, higher microbial diversity is associated with better lung function (measured by FVC and FEV_1_ z-scores) in males and increased inflammation (measured by *F*_ENO_) and lower lung function in females [15].

Yu and co-workers [16–18] showed an association between air pollution exposure and long COVID symptoms. This study combined an estimation of particulate matter ≤2.5 μm (PM_2.5_), ≤10 μm (PM_10_), black carbon and nitrogen oxide levels with an evaluation of long COVID symptoms acquired *via* questionnaires from 753 participants. Exposure to PM_2.5_ was linked to long COVID, dyspnoea and altered smell/taste [17–19]. Valencia-Hernandez *et al.* [20] presented the association of urban green spaces with lung function in children aged from 6 to 16 years in three European birth cohorts. Despite the high heterogeneity between the studies, the presence of green spaces was linked to a small increase in FEV_1_ and FVC values, although analysis of further cohorts is ongoing. Paciência and co-workers [21, 22] explained the role of exposure to air pollution as a modifier of the association between access and exposure to green spaces and development of allergic rhinitis. Their study, which included 2568 participants, demonstrated the beneficial role of green spaces, which is more important in cases of high air pollution exposure. So *et al.* [23] explained the risk of COPD and asthma in relation to air pollution. Their study investigated the Danish population and the annual mean levels of PM_2.5_, NO_2_ and black carbon. Higher exposure was related to higher asthma and COPD incidence defined by hospital contact [23–26].

J. Heinrich (Munich, Germany) presented a study on long-term exposure to ambient ozone in 3014 adults from 17 centres in nine countries [27]. Higher exposure was associated with faster lung functional decline estimated by spirometry [28, 29]. Kothe *et al.* [30] explained the impact of cooking methods on indoor air pollution and lung function in rural Rwanda. Replacing traditional cooking with improved cookstoves resulted in a reduction in indoor air pollution and an improvement in lung function. Finally, Soriano *et al.* [31] showed the estimated economic burden and carbon footprint in metric tons of CO_2_ equivalent of the change of inhalers for non-clinical reasons and the consequent lack of adherence to treatment in Spain, which is responsible for a great economic and environmental cost.

## Scientific year in review

In this “Scientific year in review” session, the speakers summarised the latest advances in translational respiratory science made by laboratories from across the world over the last year. R. Faner Canet (Barcelona, Spain) discussed the importance of gene–environment interactions in the pathogenesis of COPD. Being born severely preterm (<28 weeks gestational age) is associated with a 7-fold increased risk of developing COPD by age 30–50 years [32]. This links to another study of preterm children who underwent COPD polygenic risk scoring. Those who had the highest risk scores developed reduced FEV_1_ at the age of 5 years, which shows that COPD-associated genes may play a role in preterm children developing obstructive airways disease [33].

R. Faner Canet also discussed the role of epigenetics in COPD. A study focusing on ethnically diverse children living in low-income areas identified a genetic variant that was partly mediated by DNA methylation changes associated with smoking history. This variant was associated with reduced FEV_1_ [34]. In addition, studies continue to show the importance of telomere shortening in the development of COPD [35]. This led to an insightful conversation among the panellists about the potential to screen for individuals at risk of COPD using telomere length and polygenic risk scores.

M. Sauler (New Haven, CT, USA) presented the latest research into alveolar defects in obstructive lung disease. He explained that proinflammatory macrophages are associated with ferroptosis of alveolar type 2 (AT2) epithelial cells in lungs exposed to cigarette smoke [36]. In addition, recent data show that the loss of zinc transporter ZIP8 results in impaired AT2 cell function and subsequent lung fibrosis. Exogenous zinc then renewed the activity of AT2 cells, indicating the potential of zinc as a therapeutic target in idiopathic pulmonary fibrosis (IPF) [37]. Previous studies have shown that transfection with specific miR-200 family members (including miR-200c-3p) restores trans-differentiation of AT2 cells obtained from people with IPF to alveolar type 1 (AT1) cells [38]. More recent research showed that this is through downregulation of the endothelial Fms-related receptor tyrosine kinase 1 (Flt1) receptor [39]. Flt1 knockout mice were protected from lung fibrosis upon exposure to bleomycin, and fibrosis was even reversed in these mice [39].

M. Sauler showed research focusing on small airway disease in COPD. Single-cell RNA sequencing (scRNAseq) identified a new cell type found in distal airways, termed terminal airway-enriched secretory cells (TASCs), which secrete surfactant. There is a loss of TASCs in the distal airways of individuals with end-stage COPD, which may contribute to the loss of distal airways seen in COPD [40].

**FIGURE 2:**
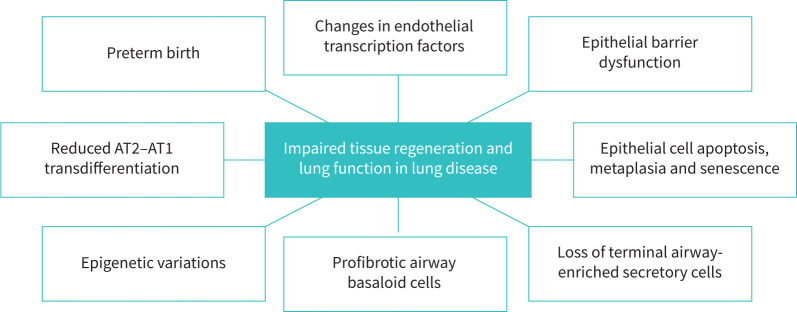
Causes of impaired tissue regeneration and lung function in lung disease. AT1/2: alveolar type 1/2.

M. Königshoff (Pittsburgh, PA, USA) focused on anti-ageing targets in IPF (figure 2). Airway basal cells in IPF are reprogrammed to a keratin 17 (KRT17)^high^ and phosphatase and tensin homolog (PTEN)^low^ cell type. These cells contributed to fibrosis development when implanted into mouse lungs, changes that were attenuated by the Src kinase inhibitor saracatinib [41]. Saracatinib, initially developed as an oncological treatment, reverses several fibrotic pathways [42].

Dysfunction of the endothelial transcription factor E-26 transformation-specific-related gene (*ERG*) occurs during ageing, and was associated with increased systemic inflammation, vascular remodelling and impaired lung fibrosis recovery following bleomycin administration [43]. Lower levels of another endothelial transcription factor, forkhead box F1 (FOXF1), were observed in endothelial cells obtained from people with IPF. FOXF1-deficient endothelial cells were associated with accelerated lung fibrosis and inflammation, and lung delivery of *FOXF1* cDNA *via* nanoparticles attenuated lung fibrosis development in mice treated with bleomycin, showing the potential of this finding as a treatment strategy in IPF [44]. These results emphasise the importance of the lung endothelium in ageing and the pathogenesis of IPF.

W. Kübler (Berlin, Germany) presented advances in the understanding of tissue barrier dysfunction in pathogen-associated respiratory failure. It has been found that the matrikine endostatin is increased in the lungs of patients with acute respiratory distress syndrome (ARDS), including COVID-19-related ARDS, and this increase promotes thrombin-induced epithelial barrier dysfunction and platelet and neutrophil activation [45]. Loss of the endothelial aryl hydrocarbon receptor (AHR) also increases tissue barrier dysfunction and subsequent movement of inflammatory cells into alveoli following influenza infection. A diet rich in AHR ligands (indoles) protects against tissue barrier dysfunction, demonstrating the importance of the gut–lung axis in viral infections [46].

W. Kübler furthermore described novel targets for pneumonia-related acute lung injury. Cystic fibrosis transmembrane conductance regulator (CFTR), the membrane channel involved in the pathogenesis of cystic fibrosis, was downregulated following *Streptococcus pneumoniae* infection. This led to endothelial barrier dysfunction through various mechanisms, including the activation of voltage-gated calcium channels and transient receptor potential vanilloid 4 (TRPV4). The CFTR potentiator ivacaftor reduced endothelial permeability following *S. pneumoniae* infection [47]. Vasculotide, agonist of the angiopoietin receptor Tie2, reduced lung permeability and acute lung injury when used with ampicillin in mechanically ventilated mice infected with *S. pneumoniae* [48].

This session highlighted the breadth and quality of translational respiratory research over the last year, covering many causes of impaired tissue regeneration and lung function in lung disease (figure 2).

## Oral presentation session: Tissue modelling and remodelling

Tissue remodelling occurs due to aberrant repair responses to tissue damage, leading to the loss of tissue integrity, disrupted extracellular matrix homeostasis and replacement with disorganised structural cells [49]. Alongside fibrosis, tissue remodelling is a common feature in many respiratory diseases, *e.g.* asthma, COPD and IPF [50]. In this session, speakers used experimental methodologies that included murine models, human *ex vivo*/*in vitro* cell culture models and single-cell-omics technologies to model diseased tissues and tease out the mechanisms underlying tissue remodelling.

### Murine models

Mutations in the surfactant protein C (SP-C) gene (*SFTPC*) in AT2 epithelial cells have been linked to sporadic and familial IPF and a fibrotic lung phenotype [51, 52]. Using a murine model of lung fibrosis where mutant SftpcI73T (I^ER^-SP-C^I73T^) was inducibly expressed, Rodriguez *et al*. [53] showed a role for epithelial metabolic dysfunction in IPF mediated by AT2 glycolytic reprogramming, mitochondrial dysfunction and altered 5′AMP-activated protein kinase (AMPK) signals which could be rescued by metformin, an indirect AMPK agonist. Next, Janciauskiene Wallmark and colleagues [54, 55] showed the beneficial effects of plasma-purified α1-antitrypsin therapy in preventing the development of obliterative bronchiolitis and attenuating acute rejection in an orthotopic model (Balb/C mice as donors and C57BL/6 as recipients) for lung transplantation.

### Human *ex vivo*/*in vitro* culture models

It has been postulated that fibroblast-derived MMPs drive extensive lung tissue destruction and remodelling during *Mycobacterium tuberculosis* (Mtb) infection [56]. Using primary human lung fibroblasts treated with control or Mtb-infected monocytes, Cusman *et al*. [57] showed that MMP-1 and MMP-3 were elevated in fibroblasts treated with Mtb-infected monocytes, and that inhibiting glycolysis with 2-deoxy-d-glucose resulted in a dose-dependent reduction in MMP-1 and reduction in *TIMP-1* gene expression. These results suggest that fibroblast MMP and TIMP-1 secretion are monocyte-dependent and indicate that host-directed strategies targeting metabolic pathways may decrease lung fibrosis in tuberculosis. Using nasal epithelial cells obtained from people with severe asthma, Soendergaard *et al.* [58] explained that people who are unable to down-titrate anti-interleukin (IL)-5 tended to have impaired wound healing (determined by a wound/scratch test), suggesting that epithelial dysfunction could be a marker of incomplete remission on treatment. In this study, complete responders to anti-IL-5 had better results on lung function tests and improved symptoms compared to non-complete responders.

This session also included work on human induced pluripotent stem cell (hiPSC)-derived lung cells, such as a study on Birt–Hogg–Dubé syndrome (BHD), a rare autosomal dominant disorder caused by germline mutations in the tumour suppressor gene *FLCN*, encoding for the protein folliculin [59]. Rodriguez Ruiz *et al*. [60] generated a BHD *in vitro* model by deleting *FLCN* in hiPSCs using CRISPR-Cas9 and differentiating those cells into iPSC-derived AT2 (iAT2) epithelial cells. Together with primary AT2 cells obtained from people with BHD, which were used to validate the *in vitro* model, the group used a lung-on-chip model to expose these cells to breathing related-stresses, as previously used for primary alveolar cell cultures [61]. Additionally, hiPSCs were used by Schweikert *et al*. [62] to generate iAT2 cells to investigate whether oestradiol affects the development of pulmonary fibrosis in non-diseased organoids. Epidemiological data on disease onset in IPF, as well as data in a bleomycin mouse model, suggest a role for sex hormones in disease pathogenesis [63]. Even though no significant differences were found in AT2 markers or selected proinflammatory or fibrotic genes in response to oestradiol, it would be interesting to further investigate the effect of sex hormones in diseased iAT2 and AT2 cells to understand potential sex-specific differences in the disease.

### Single-cell-omics

Lang *et al*. [64] observed an induction of multilineage conserved fibrogenic cell states by 1) coupling *ex vivo* cytokine and drug perturbations of human precision-cut lung slices (hPCLS) with scRNAseq to study early lung fibrogenesis directly in human tissue and 2) comparing the data against an *in vivo* multicohort single-cell atlas from pulmonary fibrosis individuals. Using micro-computed tomography (CT) staged human tissues, the authors characterised the appearance and interaction of *CTHRC1*^+^ myofibroblasts, *KRT17*^+^/*KRT5*^−^ basaloid epithelial cells and an ectopic *PLVAP*^+^/*VWA1*^+^ endothelial cell state in the thickened alveolar septum of early-stage pulmonary fibrosis. This supports the use of hPCLS for drug testing and provides a framework for in-tissue perturbational single-cell genomics [65]. Utilising a multiple iterative labelling by antibody neodeposition (MILAN) methodology on tissue sections of COPD and IPF explanted lungs, Cortesi *et al.* [66] found five distinct cell clusters (basal, AT1, AT2, intermediate AT2-to-AT1 and macrophages) based on nine phenotypic markers. They also demonstrated increased levels of leucine-rich repeat-containing G-protein coupled receptor 6 (LGR6) in basal cells, AT2 cells and intermediate alveolar progenitor populations located in fibrotic regions and in areas of inflammatory infiltration in COPD and IPF lungs that were associated with increased levels of p21 senescence marker. Next, Islam *et al*. [67] demonstrated a role for human antigen R (HuR) in lung fibroblast differentiation during IPF [68] by analysing transforming growth factor-β-treated HuR small interfering RNA (siRNA) knockdown and vector control-treated normal fibroblasts and IPF fibroblasts using concomitant RNAseq and mass spectrometry-based proteomics techniques. Lastly, Mehta *et al*. [69] provided late-breaking data from single-cell transcriptomic and T-cell receptor profiles of bronchoalveolar lavage (BAL) cells obtained from people with post-COVID-19 (>3 months from acute disease) who have residual lung abnormalities with predominantly 1) inflammatory or 2) fibrotic radiological appearance on a CT scan. They showed that the two participant groups were transcriptionally similar and exhibited clonal expansion and high T-cell receptor clustering without enrichment for SARS-CoV-2 reactive sequences, indicating that the purported radiological sub-phenotypes in such groups may well be a different manifestation of the same disease. Therefore, T-cell-directed therapies might be beneficial for these people regardless of radiological appearance.

## Hot topic: What did you always want to know about omics analyses for clinical practice?

Rapid advances in omics technologies have provided us with the tools to dissect biological processes at single-cell resolution. Integration of omics data (multi-omics) can reveal clinically important endotypes and phenotypes, with the potential to identify new therapeutic targets.

M. Nawijn (Groningen, the Netherlands) explained that the use of transcriptomics is key to understanding how cellular activity is related to its genetic information. He focused on the transition from bulk to scRNAseq, which has revolutionised pathogenesis studies by providing in-depth analysis of differences in cell-type composition, activity and (sub)phenotype within complex samples, and information on cell–cell interactions and transitions in cell state [70]. The first study presented using scRNAseq in asthma identified a novel mucous ciliated cell state, and dominance of type 2 T helper (Th2) cell signalling [71]. Further work utilising scRNAseq showed heterogeneity within Th2 cell populations and identified a subset of pathogenic IL-9-expressing Th2 cells that was increased in allergic asthmatic individuals compared to allergic individuals without asthma [72]. Strikingly, post-allergen challenge, Th2 cells were only present in the airways in asthma, and airway epithelial cells demonstrated a dramatically altered transcriptional response in subjects with asthma but not in those with allergy alone [73]. The online resource Human Lung Cell Atlas integrates multiple scRNAseq respiratory system datasets, facilitating disease comparisons at the single-cell level with the potential to identify novel targets for intervention [74].

I. Adcock (London, UK) discussed the increasing sophistication of proteomic techniques, enabling selective quantification of proteins within a complex sample. Mass cytometry by time-of-flight (CyTOF) uses heavy metal isotope-labelled antibodies to detect and quantify multiple proteins in single cells [75]. Thus, CyTOF can identify distinct cell populations, *e.g.* lung adenocarcinoma-associated immune cells [76] and various immune cell populations, in interstitial lung diseases [77]. Proteomic signatures can also be used to identify clinical phenotypes: sputum proteome clusters in asthma represent discrete molecular sub-phenotypes and identify candidate protein biomarkers [78]. It was emphasised that identifying protein “hits” requires validation over time, and it is still challenging to relate cell subtype to functionality and to demonstrate disease relevance. Using machine learning, nasal fluid protein signatures were mapped to transcriptomic datasets, identifying subsets of patients with severe asthma [79]. Further, differentially expressed gene/protein pathway analysis in this study revealed potential novel therapeutic targets.

Digital spatial profiling is a complementary technique that adds a crucial layer of information, linking transcriptomics and proteomics to imaging. F. Polverino (Houston, TX, USA) described spatial omics as a cutting-edge tool that allows structural navigation of the lung by digitally selecting regions of interest [80]. Identifying gene and protein enrichments within a specific spatial context using the same input material can predict pathologies associated with specific lung regions. The first digital spatial profiling study in COPD demonstrated that the immune checkpoint programmed death-ligand 1 (PD-L1) was spatially clustered with protein markers of activated T-cells, as well as genes involved in cancer progression. In bronchioles, PD-L1 expression was associated with functionally active alveolar macrophages and directly correlated with lung function [81].

To fully understand heterogeneity in disease, it is vital to combine multiple omics platforms. R. Faner Canet (Barcelona, Spain) illustrated different multi-omic integration approaches to inform clinical medicine. One approach is to use clinical phenotypes to identify underlying biological mechanisms (endotypes): COPD clusters, identified by spirometry and imaging, revealed differential protein and gene expression associated with distinct clinical outcomes [82]. Alternatively, multi-omics can expose mechanistic links that identify clinical phenotypes: integration of the sputum transcriptome, proteome and metabolome with the serum proteome demonstrated that airway microbiota metabolites may mediate COPD pathophysiology [83]. Ultimately, the approach used for multilevel integration depends on the research question, and the selection of platforms may influence the endotype uncovered.

In conclusion, this session highlighted the power of omics to reveal novel disease mechanisms and lead us towards precision medicine. Collaboration is vital, both for robustness and validation by increasing cohort size, and for multidisciplinary interpretation of outcomes. The challenge is to integrate clinical and multi-omic data longitudinally for therapeutic translation.

## Oral presentation session: Lost in translation: new insights into cell-to-cell crosstalk in lung disease

**FIGURE 3:**
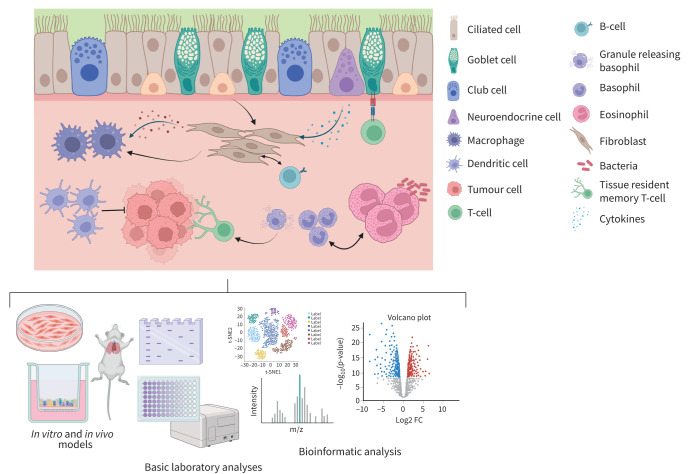
Cell crosstalk in the airways is highly complex; thus, there is a need to integrate basic laboratory techniques, multi-omics and bioinformatic analyses to holistically understand these interactions. Created with BioRender.

This session showcased how integration of next-generation sequencing with laboratory methods could be used to investigate cell-to-cell crosstalk (figure 3). First, L. De Sadeleer (Munich, Germany) introduced epithelial–mesenchymal crosstalk. This process is vital in lung regeneration and repair after injury, and of particular interest in IPF [84, 85]. Using single-nuclei RNA sequencing (snRNAseq), laser capture microdissection, spatial transcriptomics and multiplexed immunofluorescence, novel injury-associated profibrotic cell states were successfully identified. Importantly, further analysis revealed niche ligand-specific cell-to-cell interactions distinct between normal and early stages of IPF.

Next, Mansouri *et al*. [86] focused on the role of basophils in regulating tumours. Combining multilevel analyses of scRNAseq with complex laboratory models enabled an exploration of the role of understudied basophils and their interaction with regulatory T-cells (Tregs). Clinically relevant interventions (antihistamines) disturbed the interactions between basophils/Tregs, promoting tumour progression in mice. This work highlighted a surprising role of cell-to-cell crosstalk that directly impacted the risk of metastasis in humans.

A. Oliver (Cambridge, UK) shared a recent integrated cell atlas of healthy and diseased lungs [74]. The value of this database was demonstrated by combining scRNAseq and spatial transcriptomics to reveal novel circuits of cell communication between epithelial cells and CD4^+^ T-cells [87]. They highlighted increased abundance and activation of resident memory T-cells in asthma patients, an important cell type in the lung [88]. This integrated multi-omics approach identified increased interactions of goblet cells with other epithelial cells, and with CD4 T-cells, which is mediated *via* the major histocompatibility complex in people with persistent asthma. This work provides valuable insights into targetable mechanisms behind regulatory networks of T-cell activation in asthma.

Tee *et al.* [89] presented valuable insight into the anti-inflammatory role of isthmin-1 (ISM1) in allergic asthma, whose function has been described for other conditions [90, 91]. A knockout mouse model showed that ISM1 reduces eosinophil numbers in BAL fluid and reduces adiponectin secretion from AT2 epithelial cells. The multicellular effect of ISM1 deficiency directly correlated with intensified inflammation, necroptosis and airway hyperresponsiveness. This presents ISM1 as a mediator of cellular interaction and a potential therapeutic tool in allergic asthma [92].

Next, Paróczai *et al*. [93] presented findings on extracellular neutrophil traps (NETs) in airway inflammation. Charcot–Leyden crystals (CLCs), known to induce neutrophil recruitment and NET formation [94], were demonstrated to have diminished effects in complement protein-depleted mice. Granulocyte–macrophage colony-stimulating factor increased uptake of CLCs, increasing NET formation and complement proteins C3 and C5aR1. These findings reveal a novel therapeutic target in people with unresponsive asthma *via* NET-based anti-inflammatory pathways.

K. Bracke (Ghent, Belgium) used RNAseq to explore cellular crosstalk in COPD using B-cells co-cultured with fibroblasts [95]. Together with immunohistochemical co-staining for B-cells and stromal cell markers, the localisation of B-cells was discovered to affect the inflammatory and remodelling pathways of COPD, building upon previous findings [96]. As such, B-cells, lymphoid follicles and fibroblasts have dynamic roles as critical regulators of COPD [97, 98]. Zimermam *et al*. [99] explored the complex immune cell communication networks. Dendritic cells have a protective role in tumour microenvironments [100, 101]. The findings of this work show ineffective dendritic cell function in the tumoural front area. This was specific to adenocarcinoma as opposed to squamous cell carcinoma. Therefore, cell interactions are disease subtype-specific, informing therapeutic interventions.

Returning to COPD, Owles *et al*. [102] focused on IL-36γ and its effects on lung macrophages [103]. Supernatants from IL-36γ-stimulated small airway fibroblasts were exposed to monocyte-derived macrophages. Increased levels of IL-36γ impaired macrophage phagocytosis in COPD. Notably, IL-36γ expression/release is increased by viral infection [104], making this novel cell-to-cell crosstalk relevant for acute COPD exacerbations.

Finally, Lindö *et al*. [105] discussed using surgical lung tissue samples to reveal the relationship between eosinophils, microbes and immune cell patterns. Combining *in situ* hybridisation, multiplexed immunohistochemistry and spatial analysis enabled an investigation of immune infiltration patterns. No spatial correlation was found in the infiltration with bacteria, viruses or fungi. However, spatially distinct cell niches were revealed. Eosinophils and type 2 inflammatory features were linked with basophils, indicating spatial correlation [103]. This patchy pattern of immune cell niches results in a complex mix of inflammatory signatures, which impacts treatment effectiveness [106].

## Oral presentation session: From the transcriptome landscape to innovative preclinical models in lung diseases

This session highlighted a variety of state-of-the-art, innovative approaches to explore complex aspects of lung diseases, providing valuable insights into chronic airway diseases, pulmonary fibrosis, post-infection complications and conditions like COPD that result in skeletal muscle wasting.

A. Bourdin (Montpellier, France) presented a novel model combining the bronchial epithelium and submucosa, both playing an important role in many chronic airway diseases including asthma [107]. The model consists of human bronchial fibroblasts seeded on a collagen-chitosan matrix, iPSC-derived bronchial epithelial cells (forming basal, goblet, club and neuroendocrine cells) [108] and iPSC-derived neurons. To facilitate axonal integration into the existing airway epithelium, Schwann cells were added (previously described to improve nerve regeneration [109]), resulting in improved innervation of the airway epithelium that can be used to model chronic airway diseases.

Ultra-strong exercise induces physiological responses in the human body. A. Smolinska (Maastricht, the Netherlands) reported that volatile organic compounds (VOCs), which can be measured in exhaled breath using high-resolution thermo-desorption gas chromatography mass spectrometry, change after running an ultra-marathon [110]. Breath was collected pre- and post-ultra-marathon from 24 healthy participants. They found that 811 VOCs were differentially regulated, with 12 being significantly decreased and 51 significantly increased post-ultra-marathon. Seven of the significantly upregulated compounds after the ultra-marathon suggest physiological responses like fatty acid oxidation, inflammation and altered gut microbiome activity.

Lung explants mostly recapitulate the end stage of IPF, limiting the outcome of these models. Additionally, scRNAseq studies of lung explants are lacking spatial information [111–114]. A. Justet (Caen, France) applied high-resolution spatial transcriptomics to earliest clinical-grade IPF samples to recapitulate the architecture of the human airways. Early disease was characterised by a change in cell type proportions (decreased AT1, AT2 cells and general capillaries) and increased collagen XV venous and ectopic airway cells, suggesting respiratory unit loss. Thus, spatial transcriptomic analysis allows the investigation of cellular changes of the alveolar niches.

A. Valverde (Nottingham, UK) used a hiPSC-derived model with a *SFTPC* mutation generated from an individual with IPF and a CRISPR gene-edited wild-type control [115, 116] to investigate the impact of the *SFTPC* mutation on the iAT2 cell response to infection with influenza A virus subtype H1N1 [117, 118]. Bulk RNAseq revealed, in addition to top genes involved in IPF and infection, that wild-type cells mainly show Gene Ontology (GO) terms associated with the antibacterial defence response, while the mutant cell line mainly displayed GO terms associated with the cell reaction to its environment. This model demonstrates the potential of gene-edited iAT2s as *in vitro* platforms for human respiratory infection modelling.

Yamamoto *et al.* [119] presented an IPF model composed of healthy iPSC-derived alveolar organoids co-cultured with lung fibroblasts. scRNAseq showed that this model, after treatment with bleomycin, recapitulated key mechanisms of fibrosis, sharing 76.3% of upregulated pathways with IPF human-derived lung samples. Treating these fibrotic organoids with HL001, a lysophosphatidic acid 1 (LPA1) antagonist [120], showed a restorative effect with a decrease in fibrosis and an increase in AT2 cell marker. Consistent with a previous report [121], murine and human organoid models proved the effectiveness of HL001 in IPF.

By combining hPCLS generated from lung tissue from IPF donors with snRNAseq, Decaris *et al.* [122] investigated bexotegrast, a dual αVβ6/αVβ1 integrin inhibitor in the fibrotic lung models. They showed that bexotegrast reduces extracellular matrix-related gene expression in fibroblasts, attenuates the collagen triple helix repeat containing 1 (CTHRC1)^+^ pro-fibrotic fibroblast subpopulation and reduces fibrogenic gene expression pathways in aberrant basaloid cells.

Lung tissue biopsies may aid the diagnosis of fibrotic interstitial lung diseases; however, less invasive alternatives are needed. Unterman *et al*. [123] used scRNAseq to investigate novel biomarkers in BAL by characterising differences in BAL composition between fibrotic hypersensitivity pneumonitis (fHP) and IPF [124, 125]. They found that the proportions of non-fatty acid binding protein 4 (FABP4)^+^ macrophages, Tregs and C-type lectin domain containing 9A (CLEC9A)^+^ dendritic cells are significantly increased in fHP *versus* IPF. In addition, fHP macrophages showed a proinflammatory activation pattern. These findings may help to differentiate IPF from fHP without the need for invasive techniques.

Ali *et al.* [126] delved into the molecular underpinnings of the severe post-infection complication known as post-COVID pulmonary fibrosis (PCPF) [127]. This was achieved by comparing BAL samples obtained from people with PCPF with those obtained from individuals without interstitial lung disease. Analyses of Kyoto Encyclopedia of Genes and Genomes and QIAGEN Ingenuity Pathway Analysis pathways unveiled the involvement of pathways associated with the nervous system in PCPF, and identified that key regulators play a crucial role in the cytoskeleton organisation. The insights gained from this molecular investigation enhance our comprehension of PCPF and present potential therapeutic targets.

Next, Henrot *et al.* [128] explored the involvement of C-X-C motif chemokine receptor 4 (CXCR4)^+^ cells [129] in skeletal muscle wasting among people with COPD [130]. Using an early COPD mouse model with a CXCR4 deletion, the study found that this deletion prevented a decrease in muscle endurance and the loss of oxidative myofibers. P. Henrot intends to employ snRNAseq to further analyse the inflammatory infiltrate and dysregulated pathways.

Collectively, this session showcased innovative approaches in using transcriptomics (table 1) to advance our understanding of disease mechanisms and identify potential drug targets. This emphasises the significance of continued research in this field.

**TABLE 1:** Summary of the presented innovative approaches to model lung diseases presented as part of the “From the transcriptome landscape to innovative preclinical models in lung diseases” oral presentation session

**Models used**	**Transbronchial cryobiopsy**	**hPCLS**	**BAL**	**Exhaled breath**	**iPSC-derived models**	**Mouse model**
**Diseases/conditions investigated**	IPF	IPF	IPF, fHP, PCPF	Ultra-strong exercise	Asthma, IPF	COPD
**Purpose**	Investigate cellular changes of the alveolar niche in IPF [131]	Test drug bexotegrast [122]	Investigate biomarkers to characterise differences between fHP and IPF [123]; investigate molecular basis of PCPF [126]	Investigate physiological response [110]	Create novel innervated airway epithelium model [107]; model respiratory infection with H1N1 [118]; test drug HL001 [132]	Investigate role of CXCR4 in skeletal muscle wasting [128]
**Read-out**	High-resolution spatial transcriptomics	snRNAseq	scRNAseq	High-resolution TD-GC-MS	Bulk RNAseq and scRNAseq	Functional tests and whole tissue proteomics

hPCLS: human precision-cut lung slices; BAL: bronchoalveolar lavage; iPSC: induced pluripotent stem cell; IPF: idiopathic pulmonary fibrosis; fHP: fibrotic hypersensitivity pneumonitis; PCPF: post-COVID pulmonary fibrosis; CXCR4: C-X-C motif chemokine receptor 4; snRNAseq: single-nuclei RNA sequencing; scRNAseq: single-cell RNA sequencing; TD-GC-MS: thermo-desorption gas chromatography mass spectrometry.

## Concluding remarks

The selected sessions summarised in this review article showcased the diversity in basic and translational respiratory science and the remarkable progress presented at the ERS Congress 2023. The studies delved into the intricate interplay of micro- and macro-environmental factors impacting respiratory health, emphasising the urgency for comprehensive strategies addressing both environmental influences and individual behaviours. They illuminated the transformative potential of omics technologies, revealing cellular states and interactions that were previously unseen and paving the way for precision medicine. The exploration of cell-to-cell crosstalk provided deep insights into the complex networks underlying lung diseases, offering promising avenues for targeted interventions. Additionally, innovative preclinical models and advanced molecular analyses unveiled novel aspects of various lung conditions, laying the groundwork for future research and therapeutic development. The topics discussed at the ERS Congress 2023 collectively underscored the collaborative efforts and interdisciplinary approaches driving the advancements in respiratory science, offering hope for improved treatments and a healthier respiratory future for people worldwide.
